# Identification of Exhaled Metabolites in Children with Cystic Fibrosis

**DOI:** 10.3390/metabo12100980

**Published:** 2022-10-17

**Authors:** Ronja Weber, Nathan Perkins, Tobias Bruderer, Srdjan Micic, Alexander Moeller

**Affiliations:** 1 Department of Respiratory Medicine and Childhood Research Center, University Children’s Hospital Zurich, Steinwiesstrasse 75, 8032 Zurich, Switzerland; 2 Division of Clinical Chemistry and Biochemistry, University of Zurich, Steinwiesstrasse 75, 8032 Zurich, Switzerland; 3 Department of Chemistry and Industrial Chemistry, University of Pisa, Via Giuseppe Moruzzi 13, 56124 Pisa, Italy; 4 Faculty of Medicine, University of Zurich, Raemistrasse 71, 8006 Zurich, Switzerland

**Keywords:** cystic fibrosis, breath analysis, SESI-HRMS, inflammation, infection, children, putative compound identification

## Abstract

The early detection of inflammation and infection is important to prevent irreversible lung damage in cystic fibrosis. Novel and non-invasive monitoring tools would be of high benefit for the quality of life of patients. Our group previously detected over 100 exhaled mass-to-charge (*m*/*z*) features, using on-line secondary electrospray ionization high-resolution mass spectrometry (SESI-HRMS), which distinguish children with cystic fibrosis from healthy controls. The aim of this study was to annotate as many *m*/*z* features as possible with putative chemical structures. Compound identification was performed by applying a rigorous workflow, which included the analysis of on-line MS^2^ spectra and a literature comparison. A total of 49 discriminatory exhaled compounds were putatively identified. A group of compounds including glycolic acid, glyceric acid and xanthine were elevated in the cystic fibrosis group. A large group of acylcarnitines and aldehydes were found to be decreased in cystic fibrosis. The proposed compound identification workflow was used to identify signatures of volatile organic compounds that discriminate children with cystic fibrosis from healthy controls, which is the first step for future non-invasive and personalized applications.

## 1. Introduction

Chronic lung disease in cystic fibrosis (CF) is characterized by a complex interplay of inflammation and infection of the lower airways, starting already in early childhood and leading to progressing lung damage over time [1]. A timely diagnosis of bacterial infection and exacerbations is key to reducing CF-related consequences [2]. However, the dynamic processes inside the lungs are hard to monitor with the conventional methods that include the analysis of sputum or bronchoalveolar lavage fluid (BALF) [3]. Breath analysis might offer a non-invasive solution to improve the management of CF lung disease in the future.

Exhaled breath contains several hundred volatile organic compounds (VOCs) that can be related to biological processes, such as metabolism and disease-specific aspects like inflammation [4]. On-line breath analysis by secondary electrospray high-resolution mass spectrometry (SESI-HRMS) allows for the detection of a large range of mass-to-charge (*m*/*z*) features in a completely untargeted approach [5]. Previous studies showed that the method is capable of detecting putative biomarkers for respiratory diseases [6,7,8,9,10], as well as entire series of related compounds and metabolic pathways [11,12,13]. Validation and translation of the exhaled biomarkers into clinical settings represents an on-going challenge [14]. The coupling of the SESI source to a high-resolution mass spectrometer opens the door to exact compound identification of the *m*/*z* features of interest [15]. In general, compound identification is a crucial but complex and limiting step of metabolomic studies [16]. Chemical structure annotation in on-line breath analysis is challenging, due to the lack of a separation step and co-fragmentation of compounds within the quadrupole isolation window, which complicates spectral matching [17]. Methods for compound identification that have been previously applied in SESI-HRMS studies include the sampling of exhaled breath condensate (EBC) for analysis using liquid chromatography mass spectrometry (LC-MS) and subsequent fragment spectra and/or retention time matching to standard chemicals [10,13], yielding the highest degree of identification certainty. Another approach is the collection of on-line MS^2^ spectra directly from breath and a comparison to standard fragment spectra [18], or the application of semi-automated identification workflows [19,20] using software designed for the matching of fragment spectra. Lastly, some studies additionally use exact mass matching and pathway analysis for putative identification, which results in less certain annotations [18,21]. 

Several previous breath analysis studies investigated cystic fibrosis, either focusing on bacterial colonization with various pathogens [22,23], with a major focus on Pseudomonas aeruginosa infection [24,25,26], or the general characterization of CF lung disease by the comparison of patients with a healthy population [9,27,28,29,30,31,32]. Most of the described studies used GC-MS as a methodology [22,23,26,27,28,32], whereas a few used selected ion flow tube mass spectrometry (SIFT-MS) [24,25,31], SESI-HRMS [9,19,30] or laser spectroscopy [29]. Compounds that were found to distinguish patients with CF from healthy controls included increased levels of hydrocarbons, which are related to lipid peroxidation and oxidative stress [27,28], increased carboxylic acids [29], increased N-methyl-2-methylpropylamine [28] and decreased dimethyl sulfide [27]. 

This work is a follow up to a breath analysis study that was previously published by our group [30]. The study compared breath profiles acquired by on-line breath analysis (SESI-HRMS) of 52 children with CF, with 49 healthy controls (aged five to eighteen). A major result was the identification of 171 *m*/*z* features that differed significantly (adjusted *p*-value < 0.05) between the CF and control group. A total of 61 of these were elevated in patients with CF, whereas the rest were increased in the healthy children. The average predictive accuracy of the feature set to distinguish CF from healthy was 72.1%. The aim of this work was to elucidate the molecular structure of as many of these *m*/*z* features as possible, to obtain a deeper understanding of their metabolic connections and potential roles in inflammatory or pathophysiological processes.

## 2. Materials and Methods

The data used for this analysis was previously published by our group [30]. The study compared breath profiles of 52 children with CF to those of 49 healthy controls (aged 5 to 18 years) using on-line breath analysis with SESI-HRMS. A major result of our previous study was a set of 171 discriminatory *m*/*z* features (CF vs. control group). Additionally, various clinical data were collected. These results, along with the corresponding feature intensity matrix, were used as input data for this follow-up study aiming at the compound identification of the differentiating *m*/*z* features in the breath samples. The study was approved by the ethics committee of the canton of Zurich (KEK-ZH ID2017.00909), and written consent was given by the legal guardians and/or the participants.

### 2.1. Compound Identification

MS^2^ spectra were recorded directly from breath, using an on-line secondary electrospray ionization source (SuperSESI, FIT FossilionTech, Madrid, Spain) attached to a high-resolution mass spectrometer (TripleTOF 5600+, AB Sciex, Concord, ON, Canada). The methodology of measurements, material and instrumental settings were identical to the ones used for the initial comparison of CF patients and healthy controls [30]. For MS^2^ acquisition, the collisionally activated dissociation (CAD) gas was set to 6 (instead of 0 for MS^1^ acquisition), and the collision energy was set to 20 eV with a spread of ± 10 eV. The precursor isolation window of the instrument used was 0.7 ± 0.1 Da. The proprietary wiff files containing MS^1^ and MS^2^ spectra were converted to mzXML and mgf format respectively, using MSConvert (ProteoWizard 3.0.2 [33]). 

**Figure 1:**
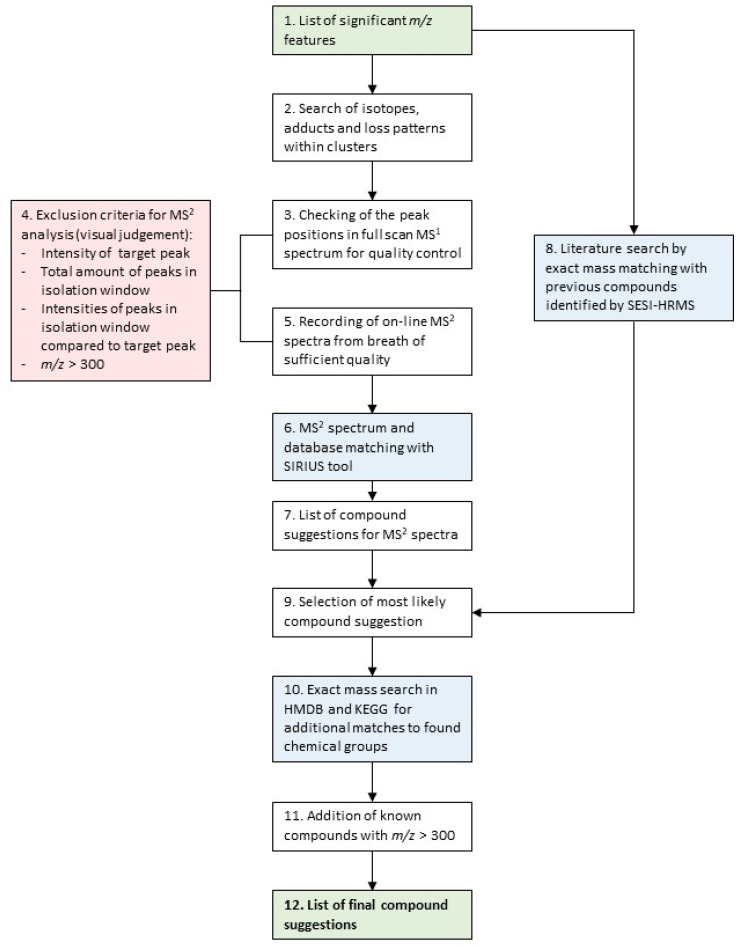
Schematic overview of the applied compound identification workflow. Details of the individual steps are described below.

The applied compound identification workflow was adapted from other publications written by our group [19,20]. An overview of the specific workflow used in this study is visualized as a flowchart in Figure 1. It is based on on-line MS^2^ fragment spectra matching with the SIRIUS software v4.9.9 [34,35,36], as well as an exact mass comparison with compounds from the SESI-HRMS literature. Finally, the Kyoto Encyclopedia of Genes and Genomes (KEGG) [37] and the Human Metabolome Database (HMDB) [38] were searched to find additional compound hits for chemical families that had more than three matches from the first two steps. The aim of this integrative and multi-step workflow was to assign the most plausible putative chemical structures to as many *m*/*z* features as possible.

Below follows a detailed description of the steps visualized in Figure 1 applied to this data set:

1. List of significant *m*/*z* features differentiating children with CF from healthy controls.
2. The *m*/*z* features were clustered in groups, according to their pairwise correlation, followed by a search of adduct and loss patterns in each group of *m*/*z* features based on differences of exact masses, as described in Kaeslin et al. [19].
3. On-line breath data acquisition was carried out sequentially by:
  - A full MS^1^ scan (*m*/*z* 50–500).
  - MS^2^ scans of the target masses.
4. The following criteria were considered, in order to decide whether or not to record an MS^2^ spectra:
  - Presence and intensity of the target peak (>30 counts per second (cps)).
  - The number of peaks (>30 cps) within the mass isolation window of 0.7 Dalton that would be co-fragmented.
  - The intensities of the surrounding peaks from b. In relation to the intensity of the target peak (intensity target peak ≥ intensity of surrounding peaks).
  - *m*/*z* features over 300 were excluded, due to the fact that their intensities were low, and the amount of possible molecular formulae and structures increases with higher masses, making the correct annotation based on on-line breath MS^2^ spectra less likely.
5. On-line MS^2^ spectra of the peaks passing the quality control were recorded.
6. The recorded MS^2^ spectra were analyzed with the SIRIUS software [34], using the following settings:
  - Molecular formula search: instrument: Q-TOF mass deviation: 20 ppm database search: KEGG and HMDB ionization: [M + H] + elemental restrictions: C, H, O, N: 0–infinity; S: 0–2
  - Structure elucidation: database search: KEGG and HMDB adducts: [M + H]+, [M + H_2_O + H]+, [M − H_2_O + H]+, [M + NH_4_]+, [M − H]−, [M + H_2_O − H]−, [M − H_2_O − H]−
7. From step 6, MS^2^ spectra received potential putative structure candidates as suggested by SIRIUS. The remaining 9 did not receive a structure suggestion.
8. In parallel, all significant *m*/*z* features were matched to compounds previously published by SESI-HRMS using exact mass comparison below a threshold of 10 parts per million (ppm).
9. The compound suggestions from the MS^2^ spectra analysis (step 6/7) and the literature comparison (step 8) were integrated to receive the most plausible final compound suggestion:
  - *m*/*z* features that only had compound hit(s) from MS^2^ analysis by SIRIUS: the putative compound suggestion with the best CSI:FingerID score [35] and protonated or deprotonated ionization, the most common ionization form in SESI [13,39], was selected. Where a different ion species had a CSI:FingerID with a magnitude of at least 30 units better than the [M + H] +/[M − H]− ionization, the corresponding putative compound suggestion was selected instead, due to the higher probability score according to the structure prediction by SIRIUS.
  - *m*/*z* features that had only a compound hit from literature: the proposed compound from the literature was selected.
  - *m*/*z* features that had hits from both the MS^2^ analysis and the literature: comparison of all possible compound suggestions from SIRIUS with that from the literature. Selection of the most plausible putative compound.
10. Additional matches to chemical groups that were represented by more than three compounds were searched by exact mass comparison with database entries in KEGG and HMDB.
11. Known compounds with high molecular weights (*m*/*z* > 300) and high intensities that were automatically excluded in step 4, were identified by exact mass comparison with a list of common mass spectrometry contaminants by Keller et al. [40] and further confirmed by on-line MS^2^ fragment spectra analysis.
12. A list of compounds that could be assigned with putative structures. The final compounds were assigned with an identification (ID) level according to Schymanski et al. [41] (ID 1 = highest certainty, ID 5 = lowest certainty). Putative compound suggestions from on-line MS^2^ analysis = ID 3, from exact mass and chemical group matching = ID 4. For compounds from the literature, the ID level was adapted to the identification certainty in the literature.

### 2.2. Data Analysis

All data analyses and visualizations were carried out in R version 4.1.1 (R Foundation for Statistical Computing, Vienna, Austria). Statistical and predictive analyses had already been performed in our previous work [30]. In addition, in order to evaluate the relationships between the metabolites, we calculated Pearson correlation coefficients for all pairs of compounds. To visualize the similarity between the identified metabolites, we used the *ComplexHeatmap* package in R [42] to perform hierarchical clustering analysis and plot the correlation matrix with dendrograms using Pearson correlation coefficients as the pairwise distance metric (distance between metabolites x and y: d = 1 − cor(x, y), where cor(x, y) is the Pearson correlation coefficient between x and y). Additionally, network modeling was used for further visualization of the relatedness among the compounds using the *qgraph* package [43]. 

## 3. Results

### 3.1. Compound Identification Workflow

As a first step, putative compound structures were suggested for the 171 significant *m*/*z* features based on the described compound ID workflow (Figure 1).

The adduct and loss relationships are listed in Table S1 (step 1). As a result of the peak quality check of the compound ID workflow (step 4), on-line MS^2^ spectra from 23 *m*/*z* features were recorded (step 5). A total of 15 received potential structure suggestions from the MS^2^ spectrum analysis with the SIRIUS software (step 6). Additionally, there were 23 compound matches based on exact *m*/*z* comparison with the SESI literature (step 8). The results from steps six and eight were then compared and integrated, to receive the most plausible putative compound suggestion (step 9). For nine *m*/*z* features, the most likely structure suggestion from the MS^2^ spectrum analysis was determined (step 9a). Only 16 *m*/*z* features had one suggestion from the literature (step 9b). Eight *m*/*z* features received compound suggestions from the MS^2^ analysis and the literature (step 9c). In seven cases, the compound suggestion from the literature was found among the possible suggestions from the MS^2^ spectra analysis, indicating that the on-line MS^2^ spectra supported the proposed compounds from the literature. In the other two cases, the most plausible suggestion from the MS^2^ spectra was chosen, due to a higher identification level than the one in the literature. Both acylcarnitines and aldehydes were frequently found among the putative compound suggestions. Ten additional acylcarnitines and one aldehyde (further supporting the suggestion from the MS^2^ analysis) were found in the KEGG and HMDB databases, based on exact mass comparison (step 10). As a last step, three significant *m*/*z* features with high molecular weights (*m*/*z* > 300) known to correspond to polysiloxanes, were added to the list of putatively identified compounds (step 11). In total, 45 *m*/*z* features were putatively identified, consisting of 34 metabolites elevated in the healthy group (Table 1) and 11 elevated in the CF group (Table 2). The column “identified based on” in Table 1 and Table 2 specifies how each individual putative compound was annotated. The type of identification (e.g., on-line MS^2^ analysis or literature) as well as the assigned ID levels reflecting the certainty of identification, are also indicated in the tables for each putative compound. 

### 3.2. Putative Chemical Structures

**Figure 2:**
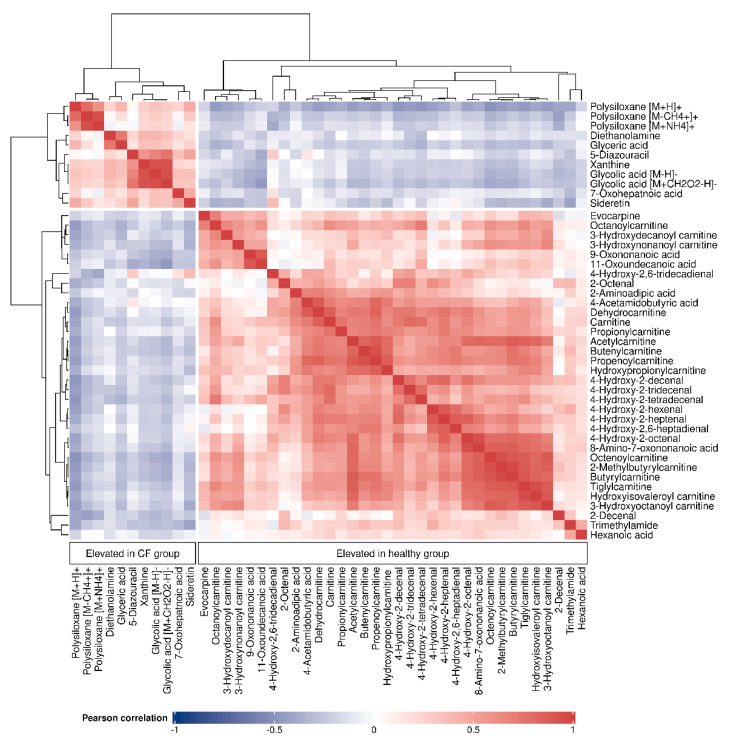
Correlation matrix heatmap of all the 45 identified compounds, with the dendrogram (right and top) depicting the relatedness among the compounds. Cell colors correspond to the Pearson correlation coefficients, ranging from blue (negative correlation) to red (positive correlation).

**Figure 3:**
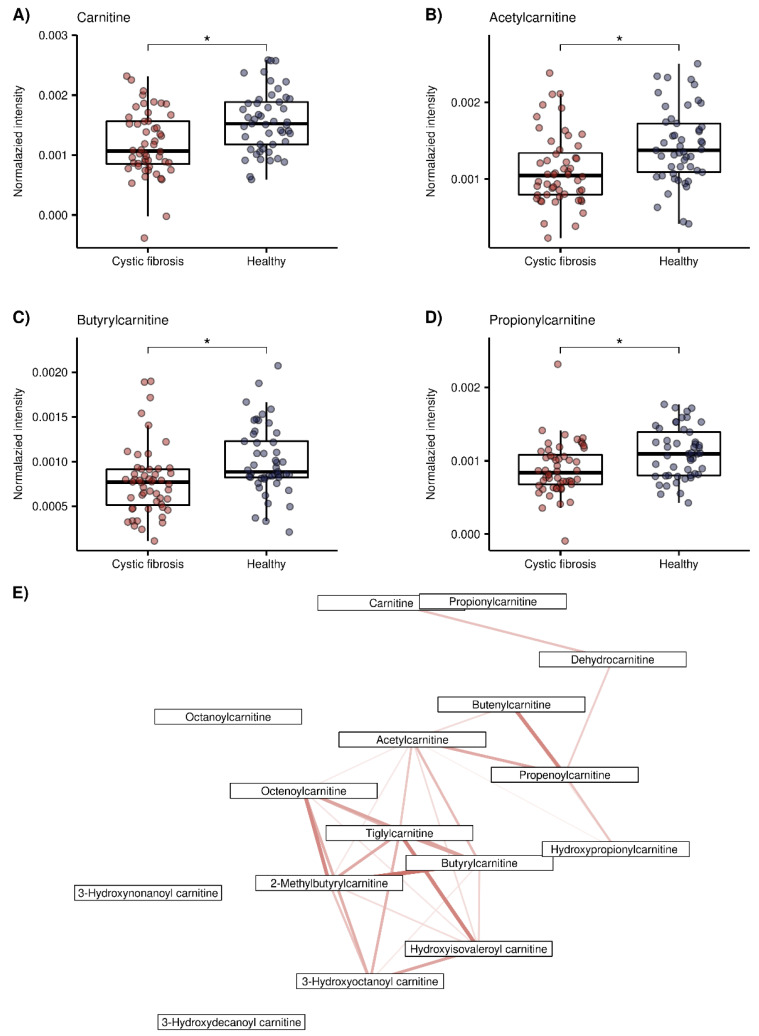
Exemplary box plots of 4 acylcarnitines (red = CF patients, blue = healthy controls), *: false discovery rate (FDR) adjusted *p* < 0.05 taken from our previous study [30]) and correlation network plot of all 16 acylcarnitines elevated in the healthy group. (**A**) Carnitine (*m*/*z* + 162.1123), (**B**) Acetylcarnitine (*m*/*z* + 204.123), (**C**) Propionylcarnitine (*m*/*z* + 218.1388, (**D**) Butyrylcarnitine (*m*/*z* + 232.154), (**E**) Correlation network plot of the 16 acylcarnitines. Edges indicate correlations between compounds, calculated using Pearson’s correlation coefficients. Red color indicates positive correlation ranging, from lighter color and thinner edges (lower correlation, minimal value = 0.78) to darker color and thicker edges (higher correlation, maximum value = 0.97). Only significant correlations (*p* < 0.05 after Bonferroni correction [46,47]) are displayed.

The relatedness between the 45 putatively identified compounds was visualized in the correlation matrix heatmap (Figure 2), with the dendrogram from the hierarchical clustering analysis. The dendrogram revealed several clusters of compounds. From the two main tree branches differentiating between metabolites elevated in the CF group (the left main branch of the top dendrogram in Figure 2) and the metabolites elevated in the healthy group (the right main branch of the top dendrogram), the next largest cluster when following the dendrogram split from top to bottom was found in the healthy group. This cluster mainly contained acylcarnitines and aldehydes. The putative compound identification workflow revealed acylcarnitines and aldehydes as the two largest groups of chemically related compounds, with 16 and 10 representatives, respectively. Figure 3 shows illustrative box plots of a selection of acylcarnitines (Figure 3A–D) as well as a correlation network plot of all the 16 acylcarnitines, visualizing the relationships between them. All 34 putatively identified compounds that were elevated in the healthy group are listed in Table 1. 

**Figure 4:**
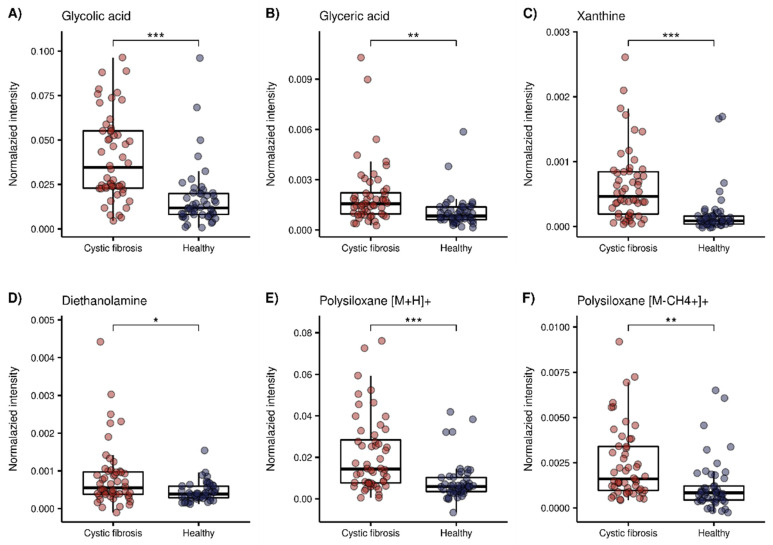
Box plots of a selection of compounds elevated in the CF group. Red = CF patients, blue = healthy controls (*: *p* < 0.05, **: *p* < 0.01, ***: *p* < 0.001 with FDR adjusted *p*-values taken from our previous work [30]). (**A**) Glycolic acid (*m*/*z* − 75.0085), (**B**) glyceric acid (*m*/*z* − 105.018), (**C**) xanthine (*m*/*z* − 151.0247), (**D**) Diethanolamine (*m*/*z* + 106.0858), (**E**) polysiloxane [M + H] + (*m*/*z* + 445.12), (**F**) polysiloxane [M-CH4 + H]+ (*m*/*z* + 429.088).

The 11 putatively identified compounds elevated in the CF group are listed in Table 2. When examining the cluster of the CF metabolites (Figure 2) the next split from top to bottom in the corresponding dendrogram showed that polysiloxanes form a cluster (left branch) which is different from several positively and negatively ionized organic compounds (right branch). Box plots of glycolic acid, glyceric acid and xanthine from the latter group are presented in Figure 4A–C. The positively ionized *m*/*z* features diethanolamine (Figure 4D), previously reported as a bacterial metabolite of Staphylococcus aureus in an in vitro study by our group [19]. The three *m*/*z* features that were annotated as polysiloxanes corresponded to forms of a single polysiloxane (dodecamethylcyclohexasiloxane) and were, as expected, highly correlated to each other, forming a distinct cluster. Two of them are visualized as box plots in Figure 4E,F.

## 4. Discussion

In this study, 45 compounds discriminating children with CF from healthy controls were putatively identified. To our knowledge, this is the largest number of compounds that have been identified in a single breath analysis study investigating children with CF.

Ten out of 45 compounds were elevated in the CF group, almost half of which were negatively ionized. Glycolic acid was detected in the deprotonated form, as well as with a formic acid adduct originating from the electrospray fluid. Glycolic and glyceric acid are structurally similar, and only differ by a hydroxyl group (Figure 4A,B). Glycolic acid is metabolized to oxalate, and increased levels of both are found in primary endogenous hyperoxaluria [48]. In CF, there is an increased incidence of secondary, absorptive hyperoxaluria and a higher risk of nephrolithiasis [49]. Since an increased glycolic acid concentration is related to primary and not secondary hyperoxaluria [48], its concentration should not be majorly affected in CF, and was therefore rarely studied. One study found increased levels of glycolic acid in urine, together with a positive correlation to oxalic acid levels in patients with CF in a targeted analysis [50], whereas an untargeted metabolomics study found no significant difference [51]. Glycerate is a metabolite of glyceraldehyde and feeds into glycolysis [52]. Elevated glycerate levels in infants with CF were found as a minor result without pathway connection, in an untargeted metabolomic study [51].

The purine base xanthine was found to be elevated in the exhaled breath of children with CF. Esther et al., reported increased levels of xanthine in the bronchoalveolar lavage fluid (BALF) of preschool children with CF to be associated with airway inflammation and structural lung disease, and a potential contributor to oxidative stress [53]. Conversely, another study found decreased levels of xanthine in CF airway epithelial cells compared with healthy cells [54]. Glycolic acid and xanthine were highly correlated (see Figure 2), although they do not seem to have an immediate metabolic relation. 

Another putatively identified compound that was increased in patients with CF was diethanolamine (Figure 4D). This compound was previously putatively identified by our group as a volatile bacterial metabolite uniquely emitted by Staphylococcus aureus, in a study of the detection of emitted VOCs from cultures of common pathogens in CF [19]. Diethanolamine was not reported as a bacterial metabolite in other studies. Of the 52 included CF patients, 37 had a positive sputum culture for Staphylococcus aureus at the time point of the breath analysis measurement. However, no significance could be found within the group of CF patients. Diethanolamine was correlated with glyceric acid, which cannot be linked to immediate biologic connections either. A reason for this might be that we do not know what degree of correlation reflects a biologically significant relation.

Dodecamethylcyclohexasiloxane, a volatile polysiloxane [55], was significantly increased in the CF group in three different ionization forms. Polysiloxanes are plasticizers, and are commonly used as additives in medical products [56], but they are also known as common mass spectrometry contaminants [40]. We hypothesize that the polysiloxane is elevated in children with CF due to the life-long and daily inhalation of medications through devices made from plastic, since previous exposure is known to be an important factor that can influence the exhaled concentration of exogenous compounds [57]. 

Out of 45 compounds, 34 were elevated in healthy children. Two large groups of chemically related compounds were found among them. The first one consisted of 16 acylcarnitines, which were either previously identified by SESI-HRMS or found by exact matching to HMDB. Additionally, carnitine itself was further confirmed by the recorded on-line MS^2^ spectrum. Figure 3A–D shows box plots of carnitine, acetylcarnitine, propionylcarnitine and butyrylcarnitine. These results are consistent with previous studies that also found decreased levels of acylcarnitines in CF [58,59,60]. O’Connor et al., reported decreased levels of acylcarnitines that correlated with inflammation and the amount of bacteria in patients with CF, as a major finding of their recent untargeted metabolomic study in BALF samples [58]. Acylcarnitines are important for cellular energy production, mainly for the β-oxidation of fatty acids, as they are used as intermediates to transport acyl groups into the mitochondria [61]. Lower concentrations of acylcarnitines in CF could therefore be potentially related to an abnormal β-oxidation of fatty acids [59]. Elevated levels of acylcarnitines have also been linked to the induction of inflammatory signaling pathways in type two diabetes, but the exact molecular targets are yet to be identified [62], and are required in order to draw potential mechanistic parallels with the pathophysiology of CF. Figure 3E visualizes the correlation network plot of the 16 acylcarnitines. Most of them have several connections to other acylcarnitines, which strengthens their annotations. 

The second group of decreased metabolites in the healthy group contained 10 aldehydes. These were mostly identified by exact mass matching with compounds from the SESI-HRMS literature. Aldehydes are known as markers of oxidative stress from lipid peroxidation [63]. Oxidative stress is known to be elevated in CF [64], which implies that aldehyde levels should also be elevated. Some studies investigated the levels of malondialdehyde (MDA) and/or 4-hydroxy-2-nonenal (4-HNE), neither of which were detected in our study in CF. Antus et al., found increased concentrations of MDA in EBC, sputum and blood plasma of CF patients [65]. Another study found elevated levels of MDA but unaltered levels of 4-HNE in the serum of CF patients [66]. Aldehydes are also environmental contaminants originating from air pollution, food and other exogenous sources, which influence the amount of exposure to aldehydes [67]. Since factors such as air pollution can have a negative effect on CF [68], patients might be more sensitized and careful in their lifestyle choices. Interestingly, another as yet unpublished study by our group also found lower levels of various aldehydes in children with asthma, compared with healthy controls [20]. 

Three omega fatty acids were found among the significant *m*/*z* features. Two of them, 9-oxononanoic acid and 11-oxoundecanoic acid were elevated in the healthy group and correlated with each other, whereas 7-oxoheptanoic acid was surprisingly increased in the CF group. However, the putatively identified 7-oxoheptanoic acid contained an H_2_O adduct and the deprotonated form was not detected, which makes its identity less certain, since all previously reported omega fatty acids were only detected in the deprotonated form [13]. The omega oxidation of fatty acids was also reported to be decreased in patients with COPD, compared with healthy controls [10]. 

The applied compound identification workflow was adapted from previous studies [19,20] and designed to assign putative compound suggestions based on on-line fragment spectra analysis and literature comparison. However, the use of on-line MS^2^ spectra and literature matching for structure annotation has some limitations. (1) The recording of on-line MS^2^ spectra often leads to co-fragmentation of several precursors within the mass isolation window of 0.7 ± 0.1 Da, which complicates the correct annotation based on fragment spectra analysis [17]. (2) We did not record fragmentation spectra by LC-MS or GC-MS to confirm the putative compound structure. (3) The *m*/*z* features were compared with those in the SESI literature. Where there was a match, the compound from the literature was selected without further confirmation, except in some cases where the compound suggestion was further confirmed by the on-line fragment spectrum. (4) Structure prediction based on the CSI:FingerID score can result in some wrong annotations, due to the large amount of chemical structures in databases [35]. The selection of the most likely putative structure selection from SIRIUS was based on fixed criteria, to reduce bias. (5) Many of the significant *m*/*z* features were of high masses (>300), which is a strength of SESI. However, such high masses represent a challenge for structure elucidation. The latter is also a reason why only 11 compounds elevated in the CF group could be putatively identified, compared with 34 in the healthy group. Apart from an imbalance in the total amount of 171 *m*/*z* features (61 elevated in the CF group and 111 elevated in the healthy group), 29 of the *m*/*z* features elevated in CF had *m*/*z* ratios over 300, compared with 19 in the healthy group, and were therefore automatically excluded from the recording of MS^2^ spectra. 

Previous breath analysis studies comparing patients with CF with a control group were either targeted studies [27], studies that analyzed the predictive power of breath profiles without compound identification [32], or studies that identified a small set of discriminatory VOCs [28,29]. In this work, we were able to detect an overall larger number of differentiating *m*/*z* features, and identify a bigger subset of exhaled metabolites than previous studies, which is advantageous to understanding more about the connections between the potential biomarkers and the pathophysiology of CF. Another difference is that this study used on-line SESI-HRMS, which yields immediate results without sample preparation. However, the predictive accuracy of 72.1% from this data set was lower than the one in Robroeks et al., which reported an accuracy for distinguishing CF patients from healthy controls by GC-MS of 100% by including 22 VOCs and of 92% by only using 10 exhaled VOCs [28]. A reason for the lower accuracy might be that we investigated a younger and healthier population of CF patients, which was reflected in forced expiratory volume in one second (FEV_1_) and body mass index (BMI) values comparable to the healthy group [30]. This might also be a reason why we could not find any significant associations between the FEV_1_, reflecting disease severity, and the significant *m*/*z* features [30]. 

In conclusion, children with cystic fibrosis exhale a distinct signature of VOCs that discriminates them from healthy controls, as measured by on-line breath analysis with SESI-HRMS. An effort was made to define a standardized compound identification workflow based on on-line MS^2^ spectrum analysis and literature matching. It was successfully applied to putatively identify 45 out of 171 exhaled compounds, some of which confirmed previous results reported in the literature and are potentially related to pathophysiology. The results also indicated that it is important to take external factors such as the inhalation of medication into account. This study was the first step towards applying on-line breath analysis as a tool to monitor CF lung disease non-invasively, by confirming that the exhaled breath of CF patients is distinct from that of healthy controls, and might directly reflect pathophysiological and/or inflammatory changes.

## Figures and Tables

**Table 1:** Compounds that were putatively identified and elevated in the healthy control group. Adj. = adjusted, Lit. = literature, ID = identification. Reproduced with permission of the © ERS 2022 [30].

No.	Mode	*m*/*z*	Adj. *p*-Value	Class	Compound	Formula	Ionization	ID Level	ID Source
1	Positive	160.0965	0.014	Acylcarnitines	Dehydrocarnitine	C7H13NO3	[M + H]+	ID4	[18]
2		162.1123	0.025		Carnitine	C7H15NO3	[M + H]+	ID1	[18], MS^2^
3		204.123	0.020		Acetylcarnitine	C9H17NO4	[M + H]+	ID1	[18]
4		216.1235	0.025		Acryloylcarnitine Propenoylcarnitine	C10H17NO4	[M + H]+	ID4	[18], HMDB
5		218.1388	0.035		Propionylcarnitine	C10H19NO4	[M + H]+	ID1	[18]
6		230.1392	0.020		Butenylcarnitine	C11H19NO4	[M + H]+	ID4	HMDB
7		232.154	0.020		Butyrylcarnitine	C11H21NO4	[M + H]+	ID4	[18]
8		234.1335	0.022		Hydroxypropionylcarnitine	C10H19NO5	[M + H]+	ID4	HMDB
9		234.1697	0.025		Tiglylcarnitine	C12H21NO	[M + H]+	ID4	HMDB
10		246.17	0.035		2-Methylbutyrylcarnitine	C12H23NO4	[M + H]+	ID4	HMDB
11		260.222	0.030		Octanoylcarnitine	C15H30NO4	[M − CO + H]+	ID4	[20]
12		262.1648	0.049		Hydroxyisovaleroyl carnitine	C12H23NO5	[M + H]+	ID4	HMDB
13		286.201	0.018		Octenoylcarnitine	C15H27NO4	[M + H]+	ID4	HMDB
14		304.2115	0.035		3-Hydroxyoctanoyl carnitine	C15H29NO5	[M + H]+	ID4	HMDB
15		318.221	0.044		3 -Hydroxynonanoyl carnitine	C16H31NO5	[M + H]+	ID4	HMDB
16		332.2448	0.048		3-Hydroxydecanoyl carnitine	C17H33NO5	[M + H]+	ID4	HMDB
17		144.138	0.005	Aldehydes	Octenal	C8H14O	[M + NH_4_]+	ID4	HMDB, MS^2^
18		172.1693	0.035		2-Decenal	C10H18O	[M + NH_4_]+	ID1	[12]
19		132.101	0.022		4-Hydroxy-2-hexenal	C6H10O2	[M + NH_4_]+	ID4	[12]
20		146.1175	0.018		4-Hydroxy-2-heptenal	C7H12O2	[M + NH_4_]+	ID4	[12]
21		160.133	0.039		4-Hydroxy-2-octenal	C8H14O2	[M + NH_4_]+	ID4	[12]
22		188.1645	0.002		4-Hydroxy-2-decenal	C10H18O2	[M + NH_4_]+	ID1	[12]
23		230.2117	0.046		4-Hydroxy-2-tridecenal	C13H24O2	[M + NH_4_]+	ID4	[12]
24		244.227	0.047		4-Hydroxy-2-tetradecenal	C14H26O2	[M + NH_4_]+	ID4	[12]
25		144.1022	0.050		4-Hydroxy-2,6-heptadienal	C7H10O2	[M + NH_4_]+	ID4	[12]
26		211.1688	0.047		4-Hydroxy-2,6-tridecadienal	C13H22O2	[M + H]+	ID1	[12]
27		60.0808	0.012		Trimethylamine	C3H9N	[M + H]+	ID3	MS^2^
31		340.2482	0.015		Evocarpine	C23H33NO	[M + H]+	ID4	[44]
28		146.0813	0.047	Acids	4-Acetamidobutyric acid	C6H11NO3	[M + H]+	ID3	MS^2^
29		162.0757	0.032		2-Aminoadipic acid	C6H11NO4	[M + H]+	ID3	[20], MS^2^
30		188.128	0.047		8-Amino-7-oxononanoic acid	C9H17NO3	[M + H]+	ID3	MS^2^
32	Negative	115.0763	0.022		Hexanoic acid	C6H12O2	[M − H]−	ID4	[45]
33		171.1028	0.014		9-Oxononanoic acid	C9H16O3	[M − H]−	ID1	[13], MS^2^
34		199.134	0.050		11-Oxoundecanoic acid	C11H20O3	[M − H]−	ID1	[13], MS^2^

**Table 2:** Compounds that were putatively identified and elevated in the CF group. Adj. = adjusted, Lit. = literature, ID = identification Reproduced with permission of the © ERS 2022 [30].

No.	Mode	*m*/*z*	Adj. *p*-Value	Class	Compound	Formula	Ionization	ID Level	ID Source
1a	Negative	75.0085	<0.001	Acids	Glycolic acid	C2H4O3	[M − H]−	ID3	MS^2^
1b		121.0143	<0.001		Glycolic acid	C2H4O3	[M + CH_2_O_2_ − H]−	ID4	Adduct/loss
2		105.0188	0.002		Glyceric acid	C3H6O4	[M − H]−	ID3	[44], MS^2^
3		151.0247	<0.001		Xanthine	C5H4N4O2	[M − H]−	ID1	[10], MS^2^
4		137.009	0.012		5-Diazouracil	C4H2N4O2	[M − H]−	ID3	MS^2^
5	Positive	106.0858	0.043		Diethanolamine.	C4H11NO2	[M + H]+	ID3	[19], MS^2^
6		163.0965	0.018		7-Oxohepatnoic acid	C7H12O3	[M + H_2_O + H]+	ID3	MS^2^
7		225.0428	0.009		Sideretin	C10H8O6	[M + H]+	ID3	MS^2^
8		429.088	0.002	Plasticizers	Polysiloxane	[C2H6SiO]6	[M − CH_4_ + H]+	ID2	[40], MS^2^
9		445.12	0.001		Polysiloxane	[C2H6SiO]6	[M + H]+	ID2	[40], MS^2^
10		462.1462	0.027		Polysiloxane	[C2H6SiO]6	[M + NH_4_]+	ID2	[40], MS^2^

## Data Availability

The data presented in this study are openly available in FigShare at https://doi.org/10.6084/m9.figshare.20601231 accessed on 1 September 2022.

## Supplementary Materials

The following supporting information can be downloaded at: https://www.mdpi.com/article/10.3390/metabo12100980/s1, Table S1 lists all 171 significant *m*/*z* features, including details from the compound identification workflow.
